# The Book World of Medicine and Science

**Published:** 1893-04-01

**Authors:** 


					viii THE HOSPITAL. April 1, 1893.
The Book World of Medicine and Science.
FIRST IMPRESSIONS OF BOOKS RECEIVED.
[It is requested that all books intended to t>9 notioed in this column
may be seat to the Eiitor, at the Offiea, 428, Strand, W.O., not later
than Tuesday evening in each week.]
Finger Prints. By Francis Galton, F.R.S. (Macmillan
and Co.)
This is a subject which Mr. Galton has made entirely his
own, and in the treatise before us he explaicB the point to
which the science of finger prints has attained, and the uses
to which it may be applied. Everyone must have noticed
the delicate tracery of lines enclosing a central aroh or loop
on the tip of each of his fingers. The extraordinary diversity
of these patterns is not, however, so easily apparent. That
no two fingers of the same person present exactly the
same conformations is a point which anyone may easily verify
for himself. The actual extent of the variation must be given
in Mr. Galton's words : " The chance of lineations which shall
be found to resemble those of a single finger print in all their
minutiae is less than one tj about sixty-four thousand
millions." In order, however, that these markings shall
serve any practical purposes of identification, it is,
of coarse, necessary to show that they are inalterable
in the individual. Mr. Galton has scrutinised prints
of the same fingers of persons of all ages taken at
intervals of many years and finds " there is absolutely no
change in say 699 out of 700 of the numerous characteristics
in the markings of the fingers of the same person." He
concludes, " there seems no persistence in any visible parts
of the body except in these minute and hitherto too much
disregarded ridges." We read of the dead body of Jezebel
being devoured by the dogs of Jezreel, so that no man might
say " This ia Jezebel," but the palms of the hands and the
soles of the feet are the very remains by which a corpse might
be most surely identified if impressions of them made during
life were available. We [learn that Sir William Herschel
employed finger prints for more than twenty years in India for
purposes of identification ; they were taken of pensioners to
prevent their personation by others after their death ; they
were used in the office for the registration of deeds, and at a
gaol where each prisoner had to sign with his fiDger. Mr.
Galton believes the use of finger prints in identifying coolies,
Hindoos, &c., whose identification by other methods seems
hopeless, would obviate all difficulties. He devotes some
space to a discussion of the best methods of taking impres-
sions of finger tips, and recommends as the simplest plan to
press them against a glass slab lightly coated with printera'
ink. The elaborate dissection of patterns illustrated by
many plates constitutes the mont valuable part of the book.
The attempt at classification of the " cores '' of the patterns
under the heads of Arches, Loops, and Whorls is a little
bewildering since the three divisions admittedly pass into
each other by almost imperceptible shades of difference.
The enormous difficulties in the way of indexing and register-
ing these minute variations have been most patiently
grappled with, but cannot be Baid to be yet overcome. Mr.
Galton devotes a chapter to the question of the hereditary
tendency in finger patterns of which he has found unmistak-
able traces. It is worth noting that the markings appear
also on the finger-tips of monkoys, and what is more remark-
able, " the prehensible tail of the howling monkey serves as a
April 1, 1893. THE HOSPITAL. ix
THE BOOK WORLD OF MEDICINE AND SCIENCE.-(continued).
fifth hand, and the naked concave part of the tail, with
which it grasps and holds on to boughs, is furnished with
ridges arranged transversely in beautiful order."
Petite Chirurgie de Jamain. (F. Alcan, Editeur, 1893.)
Jamain's Minor Surgery has for long been very popular
with the French medical student. This, the 9th edition, has
been brought well up to date by MM. Perrier and Peraire.
It contains full details of the latest aseptic and antiseptic
methods, and is a good reliable guide.
The Cardinal Symptoms of Urinary Disease, their
Diagnostic Significance and Treatment. By E.
Harry Fenwick, F.R.C.S., Surgeon to the London
Hospital. (London : J. and A. Churchill, 1893.)
A series of lectures on hcematuria, frequency of micturi-
tion, pain and abnormal urination. The original lectures
have been greatly elaborated for publication, and as the
caseB are printed in smaller, closer type, the volume has an
uninviting appearance.
A Treatise on Human Anatomy. By various authors.
Edited by Henry Morris, M.A., M.B. (Lond.), Surgeon
to Middlesex Hospital. (London : J. and A. Churchill,
1893.)
To place another large text-book of anatomy upon the
book market is a bold venture, but we anticipate a success
in this instance. The publishers have done their part well,
the paper and printing are good, and the illustrations are very
numerous (791) and admirably executed. The articles are by
well-known anatomists, and the student may rely on their
aocuracy. The volume is a very ponderous one, but with this
exception it is decidedly attractive, and that is much to say
for a book on the " dry bones " of anatomy.
The Structure and Functions of the Brain and Spinal
Cord. By Victor Horsley, B.S., F.R.C.S., F.R.S.
(Charles Griffin and Co., London, 1892.)
This is the course of Fullerian Lectures delivered in 1891,
and deals with the Spinal Cord and Medulla only. It will be
followed by other volumes on the Brain, and Physiological
Psychology. This volume opens with a review of the nervous
system of the lower animals. Needless to say, Mr. Horsley
speaks with special knowledge and authority, and the
lectures should be studied by all who desire to know the
present position of the physiology of the nervous system.
HANDY BOOKS FOR CONSULTING-ROOM TABLES.
We have received the new House of Commons, 1893,
price Is., being the thirty-ninth year of publication. It con-
tains a list of all the members of Parliament, with short
biographical notices of each member, and his address in town
and country. The book is published by Chatto and Windus,
and has a wide and deserved popularity.
The Shilling Peerage for 1893 (Chatto and Windus).?
This little book contains an alphabetical list of the House of
Lords, and much information concerning members of the
peerage and their families, and so is indispensable as a book
of reference. Complete lists of the Scotch and Irish peers
are also given, together with a short history of the Houbb of
Lords, the surnames of the peers of Great Britain and Ireland,
titles of courtesy and order of precedence amongst men and
women. Altogether this is decidedly the handiest and
cheapest peerage ever issued through the press, and what is
more, its accuracy is now universally admitted.

				

